# Anesthesia of the Patient with Zhu-Tokita-Takenouchi-Kim (ZTTK) Syndrome: A Case Report

**DOI:** 10.3390/children9060869

**Published:** 2022-06-11

**Authors:** Jan Hudec, Martina Kosinova

**Affiliations:** 1 Department of Anesthesiology and Intensive Care Medicine, University Hospital Brno, Medical Faculty of Masaryk University, 62500 Brno, Czech Republic; hudeja@gmail.com; 2 Department of Simulation Medicine, Medical Faculty of Masaryk University, 62500 Brno, Czech Republic; 3 Department of Pediatric Anesthesiology and Intensive Care Medicine, University Hospital Brno, Medical Faculty of Masaryk University, 62500 Brno, Czech Republic

**Keywords:** ZTTK syndrome, neuromuscular scoliosis, anesthesia, case report

## Abstract

Zhu-Tokita-Takenouchi-Kim (ZTTK) syndrome is an extremely rare multiorgan disorder, first described in 2015. Nowadays, about 50 patients with ZTTK syndrome have been reported, but there are no data about management during anesthesia. ZTTK syndrome patients can be indicated for surgery of musculoskeletal deformations and corrections of cardiovascular or urogenital malformations. This syndrome can be challenging for the anesthetic team based on the clinical manifestation of the syndrome. Because there are no recommendations for the management of these patients, anesthesiologists have to study typical symptoms, anatomy and possible expected changes in pathophysiology in perioperative period. One of the most dreaded anesthetic complications, the scenario “can not intubate, can not ventilate” could occur in these patients. The goal of this publication is to show options for anesthetic and perioperative management of this new rare syndrome with no published studies about management and approach in the perioperative period. The anesthetic team should choose the safest available approach. We present the first case report of anesthesia of a patient with ZTTK syndrome, a 7-year-old boy indicated for posterior neuromuscular scoliosis correction and fusion. This case describes the author’s experiences with anesthetic management and mentions possible early postoperative complications. Adequate understanding of this syndrome can reduce perioperative complications and improve patient outcomes after surgery.

## 1. Introduction

Zhu-Tokita-Takenouchi-Kim (ZTTK) syndrome is an extremely rare multiorgan disorder, first described in 2015. ZTTK syndrome represents a recent diagnosis, described in about 50 patients up to this date. Just few articles about ZTTK syndrome were published from 2015 to 2022, and most of them are focused on describing clinical and molecular findings. The authors present a unique publication from clinical practice about a patient suffering from ZTTK syndrome, because there are no published data about the perioperative management of these patients including anesthesia management. It is an autosomal dominant hereditary disorder, which affects manyorgan systems, caused by a heterozygous mutation in the SON gene (21q22.11). This mutation leads to abnormal RNA splicing [1,2,3]. The typical symptoms include intellectual development disorders, seizures, and brain malformation such ascortex malformation or corpus callosum abnormality. Facial dysmorphism includes facial asymmetry with macrocephaly, midface retrusion short philtrum, or abnormality of the nasal bridge. Musculoskeletal abnormalities are represented by hypotonia, short stature, skull abnormality, scoliosis or hemivertebrae, rib abnormalities or joint hypermobility, and contractures. Typical visceral malformations are urogenital abnormalities such as a horseshoe or unilateral kidney, cardiac abnormalities such as an atrial or ventricular septal defect, and gastrointestinal abnormalities [4,5,6]. The occurrence of these symptoms can lead to possible difficulties during perioperative management (IV line or arterial line cannulation and difficult airway management). Other rarer perioperative complications associated with this orphan disease can include the occurrence of rhabdomyolysis with hyperkalemia. This potentially lethal complication triggered by suxamethonium or volatile agents is described especially in patients with neuromuscular diseases [7]. Anesthesiologists have to study and understand these specific problems prior to any anesthesia performance.

The goal of this publication is to show possible options for anesthetic and perioperative management of this new rare syndrome with no published studies about management and approach in the perioperative period. The anesthetic team should choose the safest available approach to prevent possible adverse events. We present the first case report about the anesthesia of a patient suffering from ZTTK syndrome.

## 2. Case Presentations

Our patient is a 7-year-old boy admitted for T_4_–L_5_ neuromuscular scoliosis surgery. He weighed 14.5 kg, and he was 112 cm high, ASA physical status III.

The boy was born at full term via Caesarean section due to intrauterine growth restriction. He underwent surgery for Tetralogy of Fallot and another transventricular correction at a younger age before the ZTTK syndrome diagnosis. Detailed information about past anesthesia is not available. However, the mother did not mention significant complications. He had a psychomotor development delay with the central hypotonic syndrome. Magnetic resonance imaging showed an anomaly of the cortex and thin corpus callosum. He has suffered from hypopituitarism with hypothyroidism and epileptic seizures since 2020. His medication included levothyroxine and somatropin, as hormonal substitution, and vigabatrin was indicated due to the anamnesis of epileptic seizures. The differential diagnosis focused on genetic examination with the result of ZTTK syndrome.

**Figure 1:**
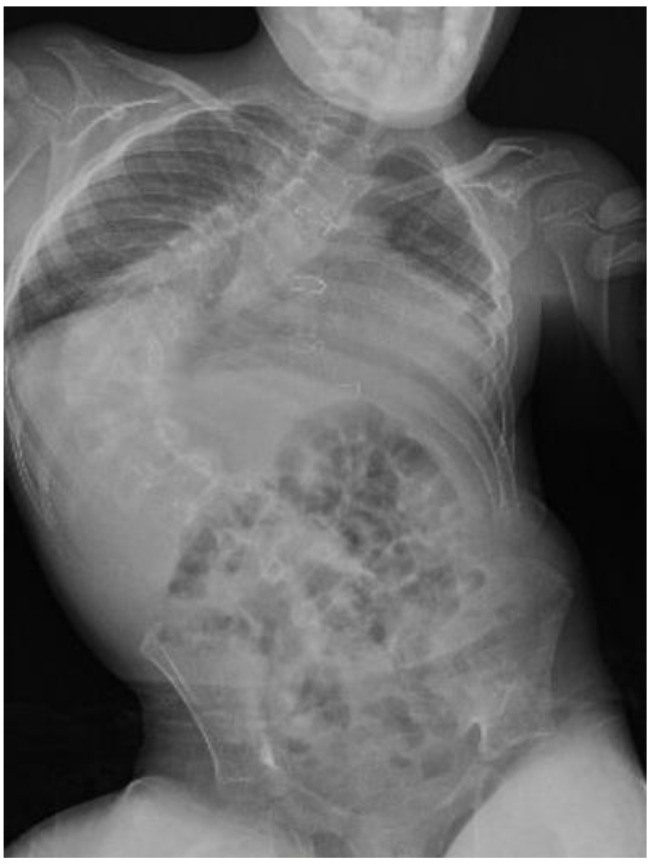
X-ray before surgery with progressive scoliosis, Cobb angle 95°.

He was able to walk with support for a short distance before the surgery. His speech was limited to a few words. Facial dysmorphism was presented by a small mouth, broad nasal bridge, and strabismus. Pulmonary functions were limited by a progressive scoliosis curve. The Cobb angle was 95° (Figure 1). Spirometry was not performed because of insufficient cooperation. ECG, echocardiography (Normal systolic function with right heart dilatation, pulmonary valve regurgitation grade III, and mild stenosis of the left branch of the pulmonary artery), and other cardiovascular findings were without contraindication to the surgery. According to the endocrinologist’s recommendation, hormone substitution with hydrocortisone, levothyroxine, and somatropin was administered. The actual EEG was without epileptic activity on the chronic medication.

In summary, our patient’s typical ZTTK syndrome abnormalities included development delay, seizures, thin corpus callosum with cortex abnormality, central hypotonic syndrome, and facial dysmorphism with a small mouth and a broad nasal bridge. Scoliosis was presented from musculoskeletal abnormalities; heart abnormalities were represented by Tetralogy of Fallot. Endocrine dysfunction included hypopituitarism with hypothyroidism.

We chose total intravenous anesthesia (TIVA) because of the monitoring of motor evoked potentials (MEP), unconvincing data about malignant hyperthermia, and the possible risk of rhabdomyolysis. We used propofol, remifentanil, and cisatracurium for anesthesia induction. We continued with propofol and remifentanil infusion. Face mask ventilation was without complications. We evaluated airways with a Cormack–Lehane score of 1 and secured airways with a cuffed endotracheal tube, size 5.0 mm. There were no complications with the intubation. After the intubation, we targeted the dose of propofol (target dose between 5–7 mg/kg/h, respectively, 70–110 mg/h) and remifentanil (target dose between 0.4–0.5 µg/kg/min, respectively, 360–450 µg/h) to a bispectral index (BIS) value between 40–60. In addition, the accelerometer was used for neuromuscular blockade monitoring before the MEP monitoring, and the patient had no residual blockade (TOF ratio above 90 %).

We turned the patient to the prone position after arterial catheter and central venous catheter cannulation. The cannulation was ultrasound-guided and uncomplicated. We used specific gel pads to prevent iatrogenic trauma. Self-warming blankets with warming pads were applied to upper and lower extremities to prevent hypothermia. Body temperature was monitored with an esophageal thermometer regularly, and the temperature did not fall below 35.9 °C. The surgery took 5.5 h. The total dose of propofol was 480 mg and the total dose of remifentanil was 1350 µg. Total blood loss was about 200 mL (13.3 mL/kg).

**Figure 2:**
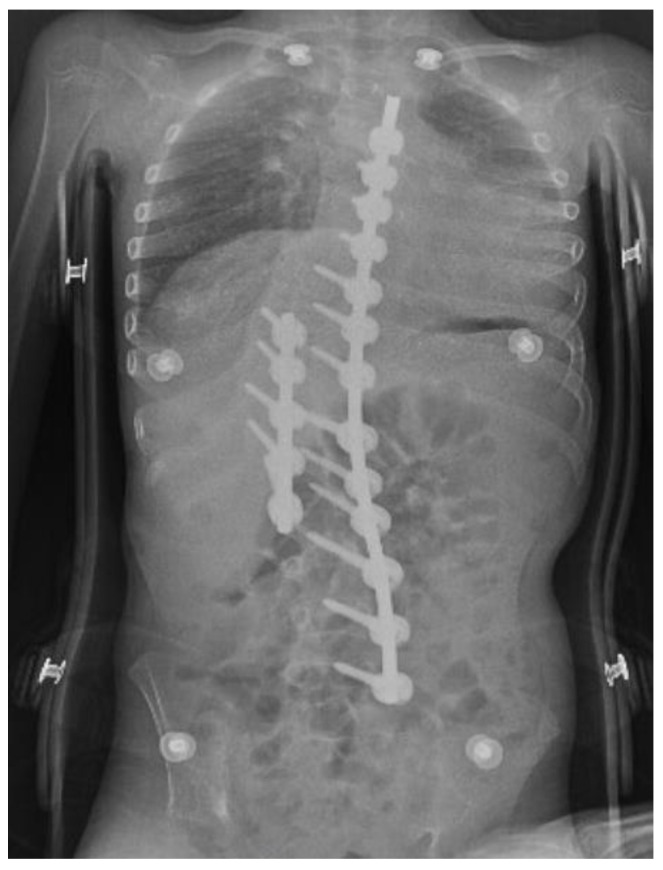
X-ray after surgery with instrumentation in the correct position.

We extubated the patient 10 min after turning him back to the supine position. The inspiratory stridor and desaturation developed after extubation. We administered oxygen via a face mask and 50 mg of hydrocortisone with sufficient reaction and further normal peripheral oxygen saturation. The inspiratory stridor duration was about 10 min. Then, the patient was admitted electively to the ICU. Early postoperative care was complicated by metabolic alkalosis and hypokalemia due to compensation of the respiratory acidosis after scoliosis correction. Potassium was substituted intravenously. This condition normalized over 10 days. Other functions normalized spontaneously. Postoperative pain management was administered with a combination of non-opioid painkillers, such as paracetamol and metamizole with a dose of 15 mg/kg IV every 6 h, and opioid therapy with piritramide with a dose of 0.1 mg/kg SC every 6 h. Regional anesthesia was not administered due to the large scoliotic curve and the potential risk of local anesthetic toxicity. The rest of the postoperative care was uncomplicated. Instrumentation was in the correct position (Figure 2). The patient was discharged home 13 days after surgery.

The 5-month follow-up after the surgery was a favorable outcome for the patient. The scoliosis was compensated with a nearly immeasurable curve. The spine instrumentation was in the correct position and the patient was without a new neurological deficit.

## 3. Discussion

To the best of our knowledge, there are no data about anesthesia and possible perioperative complications in patients suffering from ZTTK syndrome, a newly described orphan disease. As this is a newly described rare disease, it is very unlikely that strong data exist about the perioperative management of ZTTK patients due to the extremely low incidence in the population. One of the possibilities is the presentation of a rare case report focused on individual management highlighting the specifics associated with this syndrome. In this article, we reported expected challenges for the anesthetic team on one case report.

ZTTK patients can be anesthetized for the corrections of the musculoskeletal, urogenital, or cardiovascular system. Brain abnormalities and psychomotor development delay are described in most patients. Although there are no specific contraindications for regional anesthesia, general anesthesia is a method of choice in patients with limited cooperation and altered mental status [4,5].

There are no data about the risk of rhabdomyolysis or malignant hyperthermia. The incidence of malignant hyperthermia is unlikely. These two syndromes have different gene localization (chromosome 19 for malignant hyperthermia and chromosome 21 for ZTTK syndrome) [8]. However, we decided to avoid volatile agents and succinylcholine. TIVA is a safe method for the possible risk of rhabdomyolysis. Nondepolarising muscle relaxants can be used safely. Rocuronium with the available antidote sugammadex is another safe combination to avoid a prolonged neuromuscular blockade, especially in short surgeries [9]. Neuromuscular blockade monitoring is essential in patients with hypotonia, due to the risk of residual neuromuscular blockade. Urine myoglobin level monitoring can be used to diagnose rhabdomyolysis postoperatively [7,10].

Depth of anesthesia monitoring is recommended for TIVA. It shortens the time of awakening from anesthesia and reduces the risk of sedative overdosing [11]. We targeted the dose of sedative and opioid to a BIS value of 50 ± 10 to prevent overdosing. Due to proper dosing, we managed to extubate the patient 10 min after positioning him back to the supine position.

Patients with ZTTK syndrome are of short stature with small hands and short feet. Joint hypermobility, scoliosis, or contractures are described in these patients [6]. Abnormal body proportions associated with this syndrome can lead to difficult invasive access cannulation. Ultrasound-guided insertion can be helpful, especially if any anatomical deformity is present. It reduces the incidence of insertion failure [12]. In addition, body disfigurement, skull abnormalities, and hypotonia can be associated with osteoporosis. The positioning must be performed extremely carefully because of the higher risk of iatrogenic injury [13].

Mild or moderate facial dysmorphism is presented in all patients. The signs include facial asymmetry, midface retraction, short philtrum, broad nasal bridge, or small mouth with a high-arched palate. Dental abnormalities are described in the literature [4]. Although airway management was not described in the literature, these factors should lead to an expectation of difficult airway management (DAM). However, we did not have a problem with face mask ventilation and intubation. Special equipment for DAM should be prepared before every airway securing [14].

Postoperative care can be burdened by the risk of life-threatening complications in patients with neuromuscular disease, especially after major or high-risk surgery [15,16]. There is a risk of respiratory insufficiency, prolonged muscle blockade, or a potential risk of rhabdomyolysis. Vital signs in patients with ZTTK syndrome should be monitored until stabilization after surgery.

Adequate preparation and understanding of this syndrome can eliminate possible complications and improve postoperative patient outcomes. Possible anesthetic complications are mentioned in Table 1.

## 4. Conclusions

We describe the first case of anesthetic management of apatient with ZTTK syndrome. Our patient underwent total intravenous anesthesia for posterior neuromuscular scoliosis correction and fusion with no major complications. Describing this case and understanding this syndrome can help anesthetic teams to improve the perioperative management of these patients.

## Figures and Tables

**Table 1:** The frequent clinical signs and symptoms of Zhu-Tokita-Takenouchi-Kim syndrome and associated possible anesthetic complications.

Clinical Sign	Possible Anesthetic Complication
Facial asymmetry	Difficult airway management
Midface retrusion	Difficult airway management
Abnormality of the nasal bridge	Difficult airway management
Intellectual disability	Limited cooperation for invasive procedures
Development delay	Limited cooperation for invasive procedures
Neurological abnormality, hypotonia	Rhabdomyolysis
Scoliosis	Restrictive lung disease
Skull abnormality, contractures	Difficult invasive access
Contractures, joint hypermobility	Difficult positioning, iatrogenic trauma

## Data Availability

The data presented in this case report are available on request from the corresponding author.
